# Stereotactic Ablative Radio Therapy (SABR) followed by immunotherapy a challenge for individualized treatment of metastatic solid tumours

**DOI:** 10.1186/1479-5876-10-104

**Published:** 2012-05-22

**Authors:** Giuseppe V Masucci, Peter Wersäll, Rolf Kiessling, Andreas Lundqvist, Rolf Lewensohn

**Affiliations:** 1 Dept of Oncology-Pathology and KcRN, Karolinska Institutet/Karolinska University Hospital, 171 76, Stockholm, Sweden

## Abstract

Combination strategies surely play a crucial role in treatment of cancer. Stereotactic ablative radiotherapy (SABR) has been described to induce abscopal effects particularly in renal cell cancer metastases. This effect is a reaction induced following irradiation of tumour tissue and occurring in another metastatic location outside the treatment field. However, this effect is limited and occurs sparsely in about 1-5% of patient. We are planning to improve the clinical outcome of this treatment in metastatic solid tumours by combining SABR with sequential immunotherapeutic treatments including vaccination strategies, adoptive cell therapy, cytokine therapy, or anti-CTLA-4 therapy.

## Commentary

Combination strategies in treatment of human diseases in general and of cancer in particular are necessary. Combination of surgery, radiotherapy, and chemotherapy has been the standard care for treating several tumours [1,2]. In Sweden, the area of application of radiotherapy and in particular stereotactic ablative radiotherapy (SABR), has increased during the last decade [3-7]. Previous experience of SABR in the treatment of renal cell carcinoma (RCC) metastases showed a high local control (90%) at different tumor locations as documented by others and us [3-5,8,9]. The occurrence of distant micro-metastases not visualized on CT and PET remain a treatment problem. Even if the local control is high after SABR in various tumor diagnoses most patients will recur with new metastases due to the occurrence of distant micro-metastases not visualized on CT or PET/CT.

In renal cancer there have been reports on abscopal effects on distant metastases where non-irradiated tumors have regressed temporarily or seemingly permanently after treatment with SABR of either the primary tumour or other metastatic lesions [10]. The “abscopal effect” is the occurrence of objective tumor regressions induced following irradiation in sites outside the irradiated field.

This phenomenon has been reported in various tumor forms but mainly as singular events [11]. Recent evidence that radiation induces immunogenic tumor cell death and alters the tumor microenvironment to enhance recruitment of antitumor T cells supports the hypothesis that radiation can enhance both the priming and the effector-phase of the antitumor immune response [12,13]. Leukocytes phenotyping is needed to determine the underlying mechanisms for these abscopal effects. SABR treatment of inoperable renal cancer results in local tumor sterilization with release of tumour cell fragments containing molecules that may be immunogenic. For instance, apoptosis, a form of cell death originally considered as non-immunogenic and non-inflammatory, has recently been demonstrated to be immunogenic when it is induced by drugs like anthracyclines or by ionizing radiation [14,15]. These tumour antigens are taken up locally and systemically by antigen presenting cells, particularly the dendritic cells (DC), which have the potential to stimulate de novo production of specific immune responses (either cellular or humoral) or enhance, or recall already existing immune competent cells. Induction of tumor immunity to these tumor antigens is, however, regulated by cells and molecules with the ability to inhibit immune responses particularly to “self” tumor antigens. Through administration, for instance, of anti-CTLA-4 antibodies these immune regulatory mechanisms can be halted or diminished in activity, resulting in an activation of the anti-tumor responses to the antigens released by SABR, and thus acting in synergy with SABR [16].The collaborative units at our department are discussing and planning different strategies on this therapeutic combinations. Additional activation of anti-tumor immunity can also be obtained by administration of autologous DC, produced ex vivo from autologous leukapheresis derived monocytes, with the capacity to take up the circulating tumor antigens released by SABR for efficient priming of T cells. Alternatively, autologous DCs can be “pulsed” ex vivo with tumor derived material and provided as a tumor vaccine with the capacity to re-activate the patients anti-tumor response. Yet another principle of anti-tumor treatment which can be applied to these patients would be to adoptively transfer tumor specific T cells, derived either from Tumor Infiltrating T cells ( TIL ) or from autologous T cells retroviral transduced with tumor specific T cell receptors ( TCRs). SABR creates an inflammatory environment that may augment the activity of adoptively infused TILs. Consequently, we consider that the rationale beyond these strategies is:

a. to improve the clinical outcome of metastatic solid tumours (renal cell cancer, malignant melanoma, lung cancer etc) by combining the SABR with indirect induced immune re-activation;

b. to elicit an “abscopal effect” by sequential treatment with immunotherapeutic principles such as (anti CTLA-4 MoAB, autologous dendritic cells and /or TIL cells adoptive cell transfer, cytokines (GM-CSF, Interleukins etc.).

In a clinical trial recording of toxicity as well as tumour efficacy of the synergistic activity between SABR and the immune response enhancers are mandatory primary objectives. Furthermore, we intend to analyze markers that can explain possible mechanisms by establishing blood borne biomarkers for toxicity and efficacy, using LC-MS/MS; to analyse, in an unbiased way, the metabolome and proteome before and at specific times after SABR and added immunotherapy. We will elaborate the data by statistical and bioinformatics tools used at the Karolinska Biomics Centre which is part of the Eurocan Platform project . Selected protein biomarkers will be brought to a Luminex based system for analyses directed to larger materials. Recruitment of the patient cohort include at a first stage patients with metastatic renal clear cell carcinoma or malignant melanoma (Stage IV) after progression on third line therapy, with an expected survival of more than 3 months. Written informed consent will be obtained from the patient for publication of this report and any accompanying images. These patients would have at least one metastatic lesion available for SABR (15Gy x 3) and one marker lesion for evaluation of effect without SABR. In a planned phase-I study patients with metastatic solid tumors will be recruited for a combination treatment of SABR + anti-CTLA4. SABR will be delivered to at least one metastatic or primary lesion with a standard dose of 15 Gy x 3 prescribed to the 67% isodose. This dose has been documented to achieve a high local control in various tumor types and it is in accordance with experimental evidence for induction of the abscopal effect. Anti-CTLA4 will be injected i.v. with 21 days interval 1–2 days post SABR in escalating dose cohorts in order to determine the maximum tolerated dose of the antibody. Close monitoring of immunefunctions will be undertaken to identify optimal immune activating dose.

The combination of the immune triggering effects of ionizing radiation with new immune regulatory drugs may leverage the effects of radiotherapy and transform a local therapy to a novel systemic treatment.

## Authors’ contributions

All authors read and approved the final manuscript. GVM is the project leader and he has collect the material to write the commentary; PW is concentrate and specialist in SABR and has contributed to specific part of the manuscript; RK and AL are involved in preclinical immune monitoring and RL is responsible coordinator for FP7 EU grant Eurocan Platform project. All authors have conceived the study, and participated in its design and coordination and helped to draft the manuscript.

## Competing interest

The authors declare that they have no competing interests.
